# Human umbilical cord-derived mesenchymal stem/stromal cells: a promising candidate for the development of advanced therapy medicinal products

**DOI:** 10.1186/s13287-021-02222-y

**Published:** 2021-02-26

**Authors:** Miryam Mebarki, Camille Abadie, Jérôme Larghero, Audrey Cras

**Affiliations:** 1 Assistance Publique - Hôpitaux de Paris, Hôpital Saint-Louis, DMU PRISM, Unité de Thérapie Cellulaire, 1 avenue Claude Vellefaux, 75010 Paris, France; 2 INSERM CIC de Biothérapies CBT501, 1 avenue Claude Vellefaux, 75010 Paris, France; 3 Université de Paris, INSERM U976, 1 avenue Claude Vellefaux, 75010 Paris, France; 4 Faculté de Pharmacie, Université de Paris, 4 Avenue de l’Observatoire, 75006 Paris, France; 5 Université de Paris, INSERM UMR1140, 4 Avenue de l’Observatoire, 75006 Paris, France

**Keywords:** Mesenchymal stem/stromal cells, Umbilical cord, Wharton’s jelly, Advanced therapy medicinal product, Immunomodulation, Anti-inflammation

## Abstract

Umbilical cord-derived mesenchymal stem/stromal cells (UC-MSCs) emerge as a perspective for therapeutic use in immune and inflammatory diseases. Indeed, immunomodulatory and anti-inflammatory properties, associated to fewer ethical, availability, and safety issues, position UC-MSCs as a promising active substance to develop medicinal products. Since 2007, UC-MSC-based products are classified as advanced therapy medicinal products (ATMP) according to the European Regulation 1394/2007/EC. This new regulatory status required a total adaptation of stakeholders wishing to develop UC-MSC-based ATMPs. Cell production in tissue and cell banks has been replaced by the manufacturing of a medicine, in authorized establishments, according to the good manufacturing practices (GMP) specific to ATMPs. After a brief description of UC-MSCs, we described in this review their recent use in a large panel of immune and inflammatory pathologies, including early and late phase clinical trials. Despite the use of the same product, we noticed an important heterogeneity in terms of indication, posology and study design. Then, we discussed regulatory and manufacturing challenges for stakeholders, especially in terms of process harmonization and cells characterization. Our aim was to point that despite MSCs use for several decades, the development of an UC-MSC-based ATMP remains at this day a real challenge for both academic institutions and pharmaceutical companies.

## Background

Mesenchymal stem/stromal cells (MSCs) were widely described for several decades of years, with 3840 reviews referenced on the Medline/PubMed databases on November, 2020 (reviews for: mesenchymal stem cell OR mesenchymal stromal cell). Only 90 reviews concerned UC-MSCs (reviews for: mesenchymal stem cell OR mesenchymal stromal cell AND umbilical cord OR Wharton jelly). These articles can be classified into five types: (i) describing UC-MSCs isolation, characterization, and biological properties, (ii) comparing UC-MSCs with MSCs from other tissues, (iii) describing UC-MSCs clinical potential in one specific disease or (iv) a systemic analysis of all clinical trials, and (v) describing exosomes derived from UC-MSCs. However, no review has addressed the pharmaceutical development of UC-MSC-based medicines regarding regulatory aspects, manufacturing challenges and financial limits.

The aim of our review is to point the perspective that offers UC-MSCs to develop innovative cellular medicines and then to discuss challenges of such products. It is important to notice that in this review authors considered only European framework, as they are cellular therapy experts regarding European regulation. Indeed, since the publication of the European Regulation 1394/2007/EC introducing a new class of biological medicinal products, named advanced therapy medicinal products (ATMP), MSC-based products for therapeutic use must be developed according to this new status. This required a rapid adaptation of stakeholders with the necessity to transfer this biotechnology from the “stem cell graft” status to the “drug” status. In this review, authors focused on UC-MSC-based products based on their own experience. Indeed, authors contributed to the development of an UC-MSC-based ATMPs which are currently used in phase I/II clinical trials (NCT03562065 and NCT04333368).

After a brief description of these cells and their immunomodulatory properties, the most recent results of clinical trials using UC-MSCs in immune and/or inflammatory diseases are discussed. Then, we focused on regulatory definitions and guidelines that are mandatory in Europe to develop an UC-MSC-based ATMP. Manufacturing aspects were concisely reviewed. We discussed challenges for the transfer from research use to the pharmaceutical grade of UC-MSCs, according to the good manufacturing practices (GMP) specific to ATMP. Finally, we briefly discussed financial aspects that remain at this day a real challenge to make these innovative treatments available for any unmet medical need.

## Introduction

The 2000s have been marked by the emergence of a new drug category, named advanced therapy medicinal products (ATMPs). The active substance of these products can be composed of genes, cells or tissues, which differs completely from conventional medicines.

Mesenchymal stem/stromal cells (MSCs) are one of the most used cellular sources, with almost 1200 studies as reported on the ClinicalTrials.gov database on August 2020. The ease of manufacturing, absence of ethical constraints, and sufficient safety perspectives position MSCs as promising candidates to develop ATMPs. Their first therapeutic use was realized in 1995 by Arnold Caplan team [1]. Since then, there has been a significant increase of clinical trials, with only few marketing authorizations (MA). In 2012, Prochymal® (remestemcel-L) from Osiris Therapeutics, composed of allogeneic culture-expanded adult MSCs, was authorized in Canada to treat acute graft versus host disease (aGVHD) [2]. This approval was done under conditions, with a request of additional data. As of today, Prochymal® is still not marketed in Canada. In 2015, Temcell® developed by JCR Pharmaceuticals based on Osiris technology was approved for marketing and reimbursement in Japan, for the treatment of aGVHD [3]. In 2018, orphan designation was granted by the European Medicines Agency (EMA) for Obnitix® from Medac, an allogeneic bone marrow-derived MSC (BM-MSCs) product, to treat GVHD (EU/3/18/2044). The same year, EMA approved Alofisel® (darvadstrocel) from Takeda Pharma, the first MSC-derived ATMP in Europe, to treat complex anal fistulas in adults with Crohn’s disease (EMEA/H/C/004258).

These few examples show MSCs potential as drug candidates for an unmet medical need. To this day, most studies use BM-MSCs or adipose tissue-derived MSCs (AT-MSCs). Umbilical cord-derived MSCs (UC-MSCs) remain rarely used but are promising candidates as they offer several advantages compared to other sources.

## UC-MSCs definition and immunomodulatory actions

UC-MSCs are isolated from Wharton’s Jelly, a gelatinous tissue around umbilical vessels. They present many advantages compared to the gold standard BM-MSCs and  MSCs from other tissues. BM and AT harvest requires invasive procedures, under anesthesia, with risks of pain and infectious complications. Conversely, UC represents a readily available source whose collection is painless and non-invasive, with minimal ethical issues as it is usually discarded at birth. While UC-MSCs frequency is around 10^−7^% versus 10^−3^ to 10^−2^% for BM-MSCs [4, 5], they display higher proliferation capacities. Doubling time (DT) is at least two times shorter whereas the number of population doubling (PD) and clonogenicity are significantly higher [5, 6]. This can be explained by a more primitive state comparing to adult tissue-derived MSCs [7]. In addition, a long-term in vitro culture seems not to alter their phenotype and genetic stability [8]. As defined by the International Society for Cellular Therapy (ISCT®), MSCs should positively express the markers CD105, CD73, and CD90 and should not express CD45, CD34, CD14 or CD11b, CD79a or CD19, and HLA-DR [9]. Although MSCs from different tissues display similar immunophenotypic patterns based on these criteria, many studies demonstrated differences in the expression of several markers. Petrenko et al. showed that CD146 is expressed by UC-MSCs but not by AT- nor BM-MSCs, while CD133 is more expressed by BM- and AT-MSCs compared to UC-MSCs and, CD34 is expressed only by AT-MSCs [10].

**Fig. 1:**
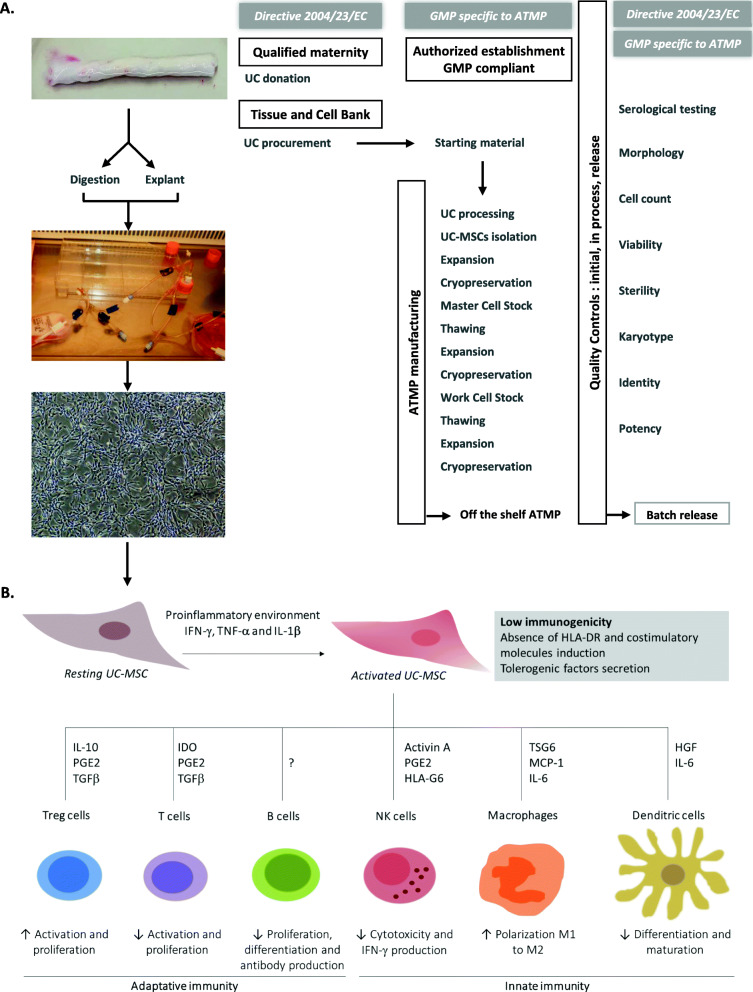
Manufacturing and mechanisms of action of an UC-MSC-based-ATMP. **a** The manufacturing of UC-MSC-based-ATMPs including the UC donation and procurement according to the European directive 2004/23/EC and then the drug manufacturing and quality controls according to GMP specific to ATMP. **b** In vitro mechanisms of action of UC-MSCs in pro-inflammatory conditions, on adaptative (left) and innate immunity (right)

The most interesting characteristics of UC-MSCs are their biological properties, in particular their ability to modulate immune responses (Fig. 1b). Even though the mechanisms of action (MOA) have not been clearly identified, both cell-to-cell contact and soluble factors are key aspects of UC-MSC-mediated properties. Immunoregulatory activities require the secretion of inflammatory cytokines by antigen-presenting cells and T cells, including interferon-*γ* (IFN-*γ*), interleukin- (IL-) 1*α*, IL-1*β*, and tumor necrosis factor-*α* (TNF-*α*) [11].

Effector T cells inhibition is the most relevant effect of UC-MSCs [12]. They induce T cells apoptosis and cell cycle arrest by secretion of indoleamine 2,3-dioxygenase (IDO) [13], prostaglandin E2 (PGE-2), and transforming growth factor-β1 (TGF-β1) [14, 15]. In addition, UC-MSCs suppress T cells activation and proliferation by altering their phenotypes and increasing the frequency of regulatory T cells [16]. These effects are mediated by the production of anti-inflammatory cytokines PGE-2, TGF-β1, and IL-10, accompanied by a decrease of the pro-inflammatory cytokine IFN-γ [16]. UC-MSCs also modulate the first step of the immune response through the inhibition of dendritic cells (DC) differentiation and maturation and the induction of monocytes towards an IL10-producing phenotype by secreting IL-6 and hepatocyte growth factor (HGF) [17]. They suppress the M1-type macrophages activation and induce the M2-type macrophages generation by TNF-α-mediated activation of cyclooxygenase-2 (COX-2) and TNF-stimulated gene-6 (TSG-6) [18]. Monocyte chemoattractant protein-1 (MCP-1) and IL-6 secretion by UC-MSCs have been shown to induce M2-type macrophages polarization [19]. Besides, UC-MSCs secrete considerable amounts of activin-A and PGE-2, which can suppress IFN-γ production by natural killer (NK) cells [20]. The cytolytic activity of NK cells is also inhibited by an immunosuppressive isoform of human leukocyte antigen-G6 (HLA-G6) produced by UC-MSCs [12]. UC-MSCs effect on B cells has poorly been investigated. One study reported the inhibition of B cell proliferation, differentiation, and antibody production [21], while another showed no inhibitory effect [22]. It is noteworthy to highlight that described MOA are based mainly on in vitro data as few in vivo studies are available concerning UC-MSCs effect on immune cells.

UC-MSCs do not generate in vitro immune responses from allogeneic T cells, indicating their low immunogenicity. This can be explained by the secretion of large amounts of tolerogenic factors including IL-10 and TGF-β1. Moreover, UC-MSCs exhibit very low expression of HLA class I, an absence of HLA-DR and co-stimulatory molecules CD40, CD80, and CD86 [12, 15, 23]. Interestingly, UC-MSCs secrete the anti-inflammatory cytokine IFN-α contrary to BM-MSCs and after an exposure to the pro-inflammatory cytokine IL-1*β* exhibit comparatively elevated expression of TGF*β*1, IDO, TSG-6, and PGE-2 [8, 24]. Upon IFN-γ stimulation, HLA-DR expression is not induced in UC-MSCs compared to BM-MSCs, suggesting a lower immunogenic profile for allogeneic use in inflammatory disease [12].

## UC-MSCs clinical applications

UC-MSCs have been tested in multiple clinical trials, with about 100 on-going studies reported on the ClinicalTrials.gov database as of September 2020. As any cell therapy, UC-MSCs present several potential risks including a short-term infectious, embolic, or acute immunogenic risks and a longer-term chronic immunogenicity or tumorigenicity [25]. In 2012, a meta-analysis of 8 clinical trials including 369 patients showed a good tolerance of MSCs [26]. In 2019, the same research group confirmed this observation with a larger meta-analysis, of 55 randomized studies, encompassing 2696 patients [27]. No study has been suspended because of serious adverse effects, confirming that MSCs are well tolerated. Even if reviewed studies differ in their indication and tissue sources, they all demonstrated MSCs safety including UC-MSCs. Wang et al. have reported a long-term safety of UC-MSCs administered in 9 patients with refractory systemic lupus erythematosus (SLE) with no hematological, liver, or cardiac side effects after 6 years [28].

Table 1 summarizes the main clinical trials using UC-MSCs in inflammatory or immune diseases including diabetes type I, SLE, multiple sclerosis, rheumatoid arthritis, Crohn’s disease, allograft-related diseases, and infections, i.e., HIV and sepsis. Several studies assessed simultaneously feasibility, safety, and efficacy. This study design is specific to ATMPs, which are generally used in few patients or even in orphan diseases. The majority of studies were randomized and controlled versus the standard of care (SOC) or a placebo. The posology was based on the patient weight ranging from 0.5 to 4 × 10^6^/kg or expressed in total cell number from 1 × 10^7^ to 3 × 10^7^ MSCs. The administration was single-dose, repeated doses, or dose escalation, using intravenous infusions the most frequently or local administrations as renal arterial injection.

**Table 1:** Overview of clinical trials using UC-MSCs in immune and inflammatory diseases

Indication	Clinical trial design	Cohort (UC-MSC/Control)	Posology	Aim	Results	Ref
Type I diabetes mellitus	Randomized, double blind, controlled (placebo)	29 (15/14)	2.6 ± 1.2 × 10^7^ Twice, 4W interval IV	Feasibility, safety, and efficacy	No acute or chronic side effects. HbA1c and C peptide improvement.	[29]
Severe and refractory systemic lupus erythematosus	Single-arm	16	1 × 10^6^/kg IV	Safety and efficacy	No treatment-related deaths. Increase in peripheral T-reg cells.	[30]
Refractory systemic lupus erythematosus	Two arms, controlled (glucocorticoids and cyclophosphamide)	37 (17/20)	3 × 10^7^ IV	Safety and efficacy	No complications. Higher serum albumin and C3, lower anti-dsDNA.	[31]
Multiple sclerosis	Randomized, controlled (AI and IS agents)	23 (13/10)	4 × 10^6^/kg 3 times 2W interval IV	Efficacy	Decrease of Expanded Disability Status Scale (EDSS) scores and relapse occurrence.	[32]
Multiple sclerosis	Phase I/phase II open-label, single-arm, single-center	20	2 × 10^7^/day 7 times IV	Feasibility, safety, and efficacy	No serious side effects. Inactive brain and cervical spinal cord lesions in 83% patients at 1 year.	[33]
Rheumatoid arthritis	Phase I/II, single-arm, single-center	64	2 × 10^7^ IV	Long-term efficacy and safety	No serious side effects at 1 year and 3 years. Decrease of disease activity score at 1 year and 3 years.	[34]
Crohn’s disease	Prospective, randomized, open- label, controlled (IS agents)	82 (41/41)	1 × 10^6^/kg, 4 times, 1W interval IV	Safety and efficacy	No serious side effects. Decrease of Crohn’s disease activity index (CDAI), Harvey-Bradshaw index (HBI), and corticosteroid dosage.	[35]
Chronic graft-versus-host disease (cGVHD) after hematopoietic stem-cell transplantation	Phase II, multicenter, randomized, double-blind, controlled (placebo)	124 (62/62)	3 × 10^7^/month 4 times maximum IV	Safety and efficacy	Side effects not associated with MSC. Decrease of cGVHD cumulative incidence at 2 years.	[36]
Acute liver allograft rejection	Randomized, controlled (IS agents)	27 (14/13)	1 × 10^6^/kg 1–3 times, 4W interval IV	Feasibility and safety	No serious side effects. Decrease of ALAT level, improvement of allograft histology.	[37]
Delayed graft function and acute rejection in deceased donor renal transplantation	Randomized, multicenter controlled	42 (21/21)	2 × 10^6^/kg IV 5 × 10^6^ renal arterial injection	Safety and feasibility	No serious side effects. Comparable graft and recipient survivals.	[38]
Severe sepsis	Phase 1, single-center, open-label, dose-escalation	15	1 × 10^6^/kg 2 × 10^6^/kg 3 × 10^6^/kg IV	Safety and feasibility	No serious side effects up to 18-months of follow-up.	[39]
HIV-1 infected immune non-responders	Pilot, randomized, open-label, controlled (HAART)	13 (7/6)	0.5 × 10^6^/kg 3 times, 4W interval, IV	Safety and efficacy	Well tolerated. Increase of naive and central memory CD4 T-cell, restoration of IFN-γ and IL-2 production.	[40]

*AI* anti-inflammatory, *IS* immunosuppressive, *IV* intravenous, *W* week

Despite the absence of phase III results, several phase I//II studies analyzed UC-MSCs efficacy as secondary endpoints. Wu et al. suggested that UC-MSCs improve haploidentical hematopoietic stem cell engraftment and reduce severe GVHD [41]. This single arm study was realized in 21 patients and need to be confirmed by a randomized and controlled study. Another phase I/II study evaluated UC-MSCs safety in multiple sclerosis and showed inactive cerebral lesions at 1 year in 83% patients [33]. More recently, UC-MSCs were introduced to manage the inflammatory symptoms of the severe acute respiratory syndrome caused by the coronavirus type 2 (SARS-Cov-2). Several clinical trials were rapidly implemented, mostly in Asia but also in the USA, South America, and Europe [35]. The majority of studies were early phases (I or I/II), some were in phase II, and none in phase III. Almost all studies were randomized and controlled versus a placebo or the SOC, two studies were not randomized but controlled, and one was a single arm. The number of subjects varied from 9 to 100. In August 2020, Shu et al. published first data of their randomized clinical trial evaluating the UC-MSCs in severe COVID-19 disease [42]. They showed a decrease of disease progression from severe to critical phase in the UC-MSCs group comparing to the SOC. More recently, Lanzoni et al. showed a significant improvement in patient survival with 91% in UC-MSCs group versus 42% in the control group [43]. Indeed, UC-MSCs can reduce the cytokine storm in COVID-19 patients, owing to their anti-inflammatory and immunomodulatory functions, which may be a crucial step in the treatment of this pathology. UC-MSCs infusion normalized lymphocytes levels and increased migration of DC to the inflammatory site [44]. Moreover, the plasma levels of C-reactive protein and pro-inflammatory cytokines, such as TNF-α and IL-6, were decreased after UC-MSCs treatment while anti-inflammatory factors such as IL-10 was increased [42–44].

## Regulatory status in Europe

The publication of the European Directive 2003/63/EC, completed by the European Regulation 1394/2007/EC introducing a new class of biological medicinal products, classified MSC-based therapies as an ATMP. ATMP definition was revised by the Directive 2009/120/EC, which classified them into four categories: (1) gene therapy medicinal products (GT), (2) somatic cell therapy medicinal products (CT), (3) tissue engineered products (TE), and (4) combined ATMPs. Since 2009, the Committee for Advanced Therapies (CAT) of EMA has delivered recommendations to classify MSC-based products as CT or TE [45]. Out of 69 MSC-based products classification requests, 21 concerned UC-MSCs with 17 products classified as TE and 4 as CT. TE are composed of UC-MSCs which are substantially manipulated and are used to regenerate, repair, or replace a human tissue. The CAT delivered recommendations to classify UC-MSCs as TE, for the treatment of intervertebral disc degeneration, burns, or non-healing wounds [45]. In the case of a CT, UC-MSCs are substantially manipulated and are administrated to treat disease through their pharmacological, immunological, or metabolic actions. Only four products are currently concerned, for the treatment of atopic dermatitis, the improvement of visual acuity in patients with vision loss from geographic atrophy secondary to age-related macular degeneration, the treatment of amyotrophic lateral sclerosis (ALS), and of acute and chronic GVHD [45].

The particularity of the ATMP development is the difficulty even the impossibility to follow a classical pharmaceutical development. Indeed, ATMPs present a large variability depending on their nature (cell, gene or tissue), their origin (autologous versus allogeneic), and the low number of patients in clinical trials. Due to the specific biologic characteristics of each ATMP, conventional pharmacological and toxicological studies may not be appropriate for the non-clinical development. Thus, non-clinical assays should take into account the nature of the product and be proportional to the expected risk in clinical use. Relevant non-clinical studies, previous clinical experience in the pathology, and pre-clinical studies could serve to the demonstration of the proof of concept and the choice of clinically endpoints for safety and efficacy evaluation. Likewise, clinical development may require alternative approaches to the classical phase I, II, and III development. Thus, a flexible approach named risk-based approach (RBA) was introduced with the revision of Annex 1, part IV of Directive 2001/83/EC as amended by Directive 2009/120/EC. This approach is optional and aims to perform a case-by-case pharmaceutical development, with the respect of scientific and regulatory guidelines relating to the quality, safety, and efficacy of medicinal products. The development of an UC-MSC-based ATMP may follow this approach in accordance with the cell therapy and/or tissue engineering guidelines:

- The guideline on human cell-based medicinal products (EMEA/CHMP/410869/2006).
- The reflection paper on stem cell-based medicinal products (EMA/CAT/571134/2009).
- The guideline on potency testing of cell-based immunotherapy medicinal products for the treatment of cancer (CHMP/BWP/271475/06).
- The reflection paper on clinical aspects related to tissue engineered products (EMA/CAT/573420/2009).
- The guideline on safety and efficacy follow-up and risk management of advanced therapy medicinal products (EMEA/149995/2008).

## Manufacturing of UC-MSC-based ATMPs

The manufacturing of UC-MSC-based ATMPs requires the donation of a human UC. UC is a human tissue and must therefore be managed in accordance with the European Directive 2004/23/EC setting standards of quality and safety for the donation, procurement, testing, processing, preservation, storage, and distribution of human tissues and cells. However, as this tissue will be used to manufacture a medicine, only donation, procurement, and testing steps are concerned. The UC correspond to the starting material of the ATMP (Fig. 1a). Involved establishments including maternities and tissue establishments must be accredited or authorized by the competent authority of each Member State. Safety tests, including serological tests, must be performed on the tissue and/or the donor. If a preservation step is required, it can also be done under the Directive 2004/23/EC. The rest of the manufacturing must be in accordance with the Directive 2001/83/EC and the guidelines on GMP specific to ATMP (GMP-ATMP). UC-MSCs can be isolated by two main methods, the explant method and the enzymatic digestion method, from several compartment of the UC (Fig. 1a) [46]. Briefly, the explant method consists of cutting the UC into fragments of few centimeters named explants, which are seeded in a plastic culture supports for 7 days. When MSCs reach 80% confluence, they are detached using a trypsin solution, and then expanded in a higher surface culture, for one or several passages, until reach the suitable cell quantity [47]. During the enzymatic digestion method, the UC fragments are exposed to enzymatic solution, using manual or automatized agitators. This method offers the possibility to isolate cells in a closed system; however, it requires the use of GMP-compliant enzymes and equipment [48]. Regardless the protocol, the frequency of isolated MSCs is still very low, requiring one or several expansion steps to produce a sufficient quantity for clinical use. The number of required expansion steps is expressed by the number of cell passage, DT, and PD, the latter two being more informative. These parameters widely vary between manufacturers, showing the complexity to standardize manufacturing processes [49]. Indeed, cell expansion is impacted by several factors including culture media, atmospheric conditions (oxygen tension 21% versus 5%), and cryopreservation. Regarding the culture media, fetal bovine serum (FBS) supplemented media has been the gold standard for a long time. However, GMP processes recommend to use raw materials of pharmaceutical grade and consequently free of xenogeneic compounds. Particularly, compliance with the guideline on minimizing the risk of transmitting animal spongiform encephalopathy agents is required (EMA/410/01 rev.3). When possible, the used raw materials should meet the European Pharmacopoeia guidelines on raw materials of biological origin (Eur. Phar. General Chapter 5.2.12). In recent years, several GMP-compliant media were developed to replace the FBS. The most commonly used is still human platelet lysate supplemented medium [49]. However, an increasing development of ready-to-use media was observed, such as MSC NutriStem® XF (Biological Industries), MesenCult™ MSC (Stemcell Technologies), Prime-XV MSC Expansion (Irvine Scientific), and StemMACS™ MSC (Miltenyi Biotec).

During the manufacturing, one or several cryopreservation steps of culture-expanded UC-MSCs can be performed. As UC is an unlimited source, it offers the possibility to generate Master Cell and Work Cell Stocks to facilitate the manufacturing and to produce several batches from one donor. The use of bioreactors for such manufacturing will help to implement reproducible and standardized procedures, which is a challenge for ATMPs.

Quality controls (QCs) must be performed according to the clinical trial authorization or the MA (Fig. 1a). To this day, there is no consensus on QCs to be performed on an MSC-based-ATMP, but they should include at least tests of identity, purity, safety, and potency. The identity and purity are conventionally carried out by a flow cytometry immunophenotyping based on the membrane markers defined by the ISCT®. It should be noted that the ISCT® minimal criteria are proposed as recommendations and are not to be confused with the medicine release specifications. Specifications are established during the pharmaceutical development of the ATMP, in accordance with regulatory directives and guidance and may vary between manufacturers. As an example, UC-MSCs phenotype may be influenced by culture conditions and the number of passages [50]. An assessment of cell viability and morphology is also mandatory as well as their growth kinetic by calculating the number of PD and the DT. Product safety concerns sterility which includes microbiology, mycoplasma, and endotoxin testing and the maintenance of genomic stability during in vitro expansion by karyotype analysis, comparative genomic hybridization array, or fluorescence in situ hybridization. The bioactivity, assessed by potency tests, may also vary depending on the manufacturing conditions or on the route of administration. Indeed, it will influence the cell microenvironment which will affect their functionality. Potency tests must take into account the clinical indication and the expected MOA. In 2013, the MSC Committee of the ISCT® has published a proposal for a standardized approach to characterize MSC immunomodulatory properties [51]. Among the suggested tests appears the assessment of MSC immune plasticity after a pro-inflammatory priming with IFN-γ and TNF-α. In order to standardize practices, the ISCT® recommended in 2016 a collegial work to an open access for all potency assays validated by regulatory authorities [52].

Beyond the manufacturing and QCs, the use of GMP- compliant facilities and equipment with an environmental control are required. Personnel must be trained and qualified to work with GMP compliance and a qualified person is designed for batches release. Finally, a robust quality management system with a strong traceability system is key element of the entire pharmaceutical circuit.

## MSC-based ATMPs cost

MSC-based products are among the less expensive ATMPs. UC-MSCs offer the possibility to be even cheaper thanks to the unlimited starting material which allows to produce unlimited number of cell banks. These off-the-shelf products, readily available, allow to reduce costs comparing to others ATMPs. As an example, granted chimeric antigen receptor T cell (CART cell) gene therapies Kymriah and Yescarta cost around 350,000€ per patient. Luxturna, another gene therapy product for the treatment of inherited retinal dystrophy, costs around 800,000€ per treatment. Zolgensma, a gene therapy indicated for treating spinal muscular atrophy, was authorized in Europe in 2020. To this day, it is the most expensive therapy in the market, priced at 1.9 million € per patient. MSC-based ATMPs are significantly cheaper with the example of Alofisel costing only 54,000€ per treatment in France [53]. Temcell, another MSC-based product, was approved for reimbursement in Japan at a maximum price of 104,000$ per treatment [3]. Mastrolia et al. estimated the manufacturing of allogeneic MSC-based medicines from 15,000 to 30,000€ per patient [54]. These data are in accordance with our own estimation of a UC-MSC-based-ATMP manufacturing as part of the clinical trial NCT04333368.

## Conclusion

UC-MSCs are strongly expanding candidates to develop ATMPs, for a high unmet medical need in the field of immune and inflammatory diseases. Indeed, a rapid expansion of clinical trials was observed these past few years. However, manufacturing standardization, product characterization, and limitation of inter-donor variabilities remain challenges for MA granting. Regulatory guidelines on the development of cell-based medicinal products associated to the ISCT® recommendations offer solid basis for stakeholders to develop UC-MSC-based ATMPs. Yet, these guidelines are not to be confused with specifications that must be validated by each developer and which are therefore heterogeneous. Implementation of a consensus to standardize manufacturing process and product qualification appears necessary but is still a debated question. Indeed, a classic and standardized pharmaceutical development remains difficult or even impossible. A best knowledge of MSCs MOA and the improvement of their characterization are critical challenges to advance the development of these products.

Moreover, MSCs have shown a good safety profile but still need more efficacy data that appear heterogeneous. Indeed, clinical effect can be influenced by several factors including donors, tissue source, cell quantity and/or bioactivity, manufacturing process, or route of administration. As a standard clinical development appears difficult, regulatory considerations will be adapted to each medicine regarding the RBA and the risk/benefit balance. Additional requests may be asked by regulatory authorities to complete the MA dossier. However, regarding the unmet medical need, holders can be granted conditional MA even if efficacy data are judged incomplete or insufficient. Indeed, the innovative character of ATMPs offers the possibility to complete these data during clinical trials realized after the granting of conditional MA. Depending on supplemental data, the conditional MA can be validated or withdrawn.

ATMPs present a high rate of conditional MA, MA under exceptional circumstances and failed MA [54]. Experimental and clinical design represented significant objections during regulatory assessments reflecting the lack of ATMP non-clinical and clinical standardizations. Despite a relatively easy manufacturing process comparing to genetically modified cell-based ATMPs (i.e., CART-cells) and a lower cost, many efforts are still needed to grant MA to an UC-MSC-based ATMP.

Several MSC-based medicines (BM-, AT-, or UC-MSCs) have been granted MA, which remain limited to certain countries. In Europe, only one MA was granted, Alofisel®, an allogeneic AT-MSC-based CT product, to treat complex anal fistulas in Crohn’s disease. Prochymal®, an allogeneic BM-MSC-derived medicine, was approved in Canada and New Zealand for the treatment of GVHD [55]. Stempeucel, a second allogeneic BM-MSC-derived medicine would be approved in India to treat critical limb ischemia (https://www.stempeutics.com/). Several MSC-based medicines were authorized in South Korea including Neuronata-R® and Cellgram®, two autologous BM-MSC-based medicines, for the treatment of amyotrophic lateral sclerosis and acute myocardial infarction respectively. In addition, Cupistem® an autologous AT-MSC-based medicine was approved for the treatment of Crohn’s fistula and Cartistem® an allogeneic UC-MSC-derived medicine for the treatment of knee cartilage defects in patients with degenerative or repetitive traumatic osteoarthritis [56].

It is important to highlight that nowadays, no UC-MSC-derived medicine has been authorized for the treatment of immune and/or inflammatory diseases, offering several perspectives to stakeholders.

## Data Availability

Not applicable.

## Abbreviations

AT: Adipose tissue
AT-MSCs: Adipose tissue-derived MSC
aGVHD: Acute graft versus host disease
ATMP: Advanced therapy medicinal products
BM: Bone marrow
BM-MSCs: Bone marrow-derived MSC
CART-cells: Chimeric antigen receptor T cells
CAT: Committee for Advanced Therapies
COX-2: Cyclooxygenase-2
CT: Somatic cell therapy medicinal products
DC: Dendritic cells
EMA: European Medicines Agency
FBS: Fetal bovine serum
GMP-ATMP: Good Manufacturing Practices specific to ATMP
GT: Gene therapy medicinal products
HGF: Hepatocyte growth factor
HLA-G6: Human leukocyte antigen-G6
IDO: Indoleamine 2,3-dioxygenase
IFN-*γ*: Interferon-*γ*
IL: Interleukin
ISCT: International Society for Cellular Therapy
MA: Marketing authorizations
MCP-1: Monocyte chemoattractant protein-1
MOA: Mechanisms of action
MSCs: Mesenchymal stem/stromal cells
NK: Natural killer
PD: Population doubling
PGE-2: Prostaglandin E2
QCs: Quality controls
RBA: Risk-based approach
SARS-Cov-2: Severe acute respiratory syndrome caused by the coronavirus type 2
SLE: Systemic lupus erythematosus
SOC: Standard of care
TD: Time doubling
TE: Tissue engineered products
TGF-β1: Transforming growth factor-β1
TNF-*α*: Tumor necrosis factor-*α*
TSG-6: TNF-stimulated gene-6
UC: Umbilical cord
UC-MSCs: Umbilical cord-derived mesenchymal stem/stromal cells
